# Multi-Omics-Based Identification and Functional Characterization of *Gh_A06G1257* Proves Its Potential Role in Drought Stress Tolerance in *Gossypium hirsutum*

**DOI:** 10.3389/fpls.2021.746771

**Published:** 2021-10-21

**Authors:** Teame Gereziher Mehari, Yanchao Xu, Muhammad Jawad Umer, Margaret Linyerera Shiraku, Yuqing Hou, Yuhong Wang, Shuxun Yu, Xianlong Zhang, Kunbo Wang, Xiaoyan Cai, Zhongli Zhou, Fang Liu

**Affiliations:** ^1^State Key Laboratory of Cotton Biology, Cotton Institute of the Chinese Academy of Agricultural Sciences, Anyang, China; ^2^National Key Laboratory of Crop Genetic Improvement, Huazhong Agricultural University, Wuhan, China; ^3^School of Agricultural Sciences, Zhengzhou University, Zhengzhou, China

**Keywords:** transcriptome, metabolome, *Gossypium hirsutum*, WGCNA, overexpression

## Abstract

Cotton is one of the most important fiber crops globally. Despite this, various abiotic stresses, including drought, cause yield losses. We used transcriptome profiles to investigate the co-expression patterns of gene networks associated with drought stress tolerance. We identified three gene modules containing 3,567 genes highly associated with drought stress tolerance. Within these modules, we identified 13 hub genes based on intramodular significance, for further validation. The yellow module has five hub genes (*Gh_A07G0563, Gh_D05G0221, Gh_A05G3716, Gh_D12G1438*, and *Gh_D05G0697*), the brown module contains three hub genes belonging to the aldehyde dehydrogenase (ALDH) gene family (*Gh_A06G1257, Gh_A06G1256*, and *Gh_D06G1578*), and the pink module has five hub genes (*Gh_A02G1616, Gh_D12G2599, Gh_D07G2232, Gh_A02G0527*, and *Gh_D07G0629*). Based on RT-qPCR results, the *Gh_A06G1257* gene has the highest expression under drought stress in different plant tissues and it might be the true candidate gene linked to drought stress tolerance in cotton. Silencing of *Gh_A06G1257* in cotton leaves conferred significant sensitivity in response to drought stress treatments. Overexpression of *Gh_A06G1257* in Arabidopsis also confirms its role in drought stress tolerance. L-valine, Glutaric acid, L-proline, L-Glutamic acid, and L-Tryptophan were found to be the most significant metabolites playing roles in drought stress tolerance. These findings add significantly to existing knowledge of drought stress tolerance mechanisms in cotton.

## Introduction

Cotton (*Gossypium* spp.) has been cultivated for many years by human beings (Fang et al., 2017). Today, *G. hirsutum* accounts for 95% of the yearly cotton production globally (Ullah A. et al., 2017) with India, China, United States, Pakistan, and Brazil being the leading five cotton-growing countries in the world. They produce 76% of all cotton on the globe (Baytar et al., 2018). China's cotton industry has particularly grown considerably in the last 60 years. It produces 30% of the world's cotton despite only having 15% of acreage for cotton at present (Dai and Dong, 2016).

Drought stress causes extensive crop loss and is predicted to intensify in the future. As a result, a global movement is underway to promote drought-tolerant crops (Shadakshari and Shanthakumar, 2015). Drought tolerance occurs as a result of a chain of molecular, cellular, and physiological developments including the induction and/or repression of a variety of genes that are the basis of the buildup of numerous osmolytes, enhanced antioxidant system, decreased transpiration, repressed shoot growth, and decreased tillering (Joshi et al., 2016). Plants have several mechanisms to conquer this abiotic stress such as drought avoidance (i.e., reduction of transpiration by closing stomata and thus bearing inner water potential), drought escape (i.e., rapid maturation), and drought tolerance (i.e., dealing with water stress without changing physiological features) (Iqbal et al., 2013). Plants change the transcriptional activity of stress response genes at the molecular level in response to abiotic stimuli.

Omics techniques have been shown to account for the majority of relevant and prospective biotechnological tools for enhancing plant abiotic stress tolerance (Bagati et al., 2018). Transcriptome has been broadly employed to investigate how stress factors impact the transcriptome of crops. As a result, the ability of a crop to maintain photosynthesis in the face of drought is a substantial measure of drought tolerance. However, the expressions of these genes in response to drought stress tolerance remains poorly studied (Shi et al., 2018). It is a decisive tool that offers a combined task of the genes along with information on gene abundance and expression levels in various plant tissues when crops are faced with numerous abiotic and biotic stress elicitors (Magwanga et al., 2018).

In addition, metabolomics displays crucial secondary metabolites of tolerant varieties for fighting abiotic stress (Fahimirad and Ghorbanpour, 2019). Metabolites are believed to be signaling molecules because they are correlated with physiological practices and are distributed from each organelle to the cytoplasm in the form of retrograde signals. Plant responses to drought are influenced by interactions between genes, metabolites, proteins, and the drought-responsive transcriptome. As a result, by integrating transcriptome and metabolomics methods, we can better recognize the processes underlying fundamental plant resistance to drought stress. Many studies on the integration of transcriptome and metabolome have been reported in response to salt stress in *Astragalus membranaceous*, foxtail millets, and sesame (Jia et al., 2016; Shi et al., 2018; Zhang et al., 2019), whereas some used integrated approach in *Nicotiana tabacum* and rice under cold stress (Ma et al., 2016; Jin et al., 2017).

Progress in functional genomics of cotton will depend on enlarging high-throughput technologies and integrating multidisciplinary action toward future cotton enhancement programs (Ashraf et al., 2018). Moreover, Abdelrahman et al. (2015) stated that for a long time, breeding and continuous cultivation for desired agronomic features has had a negative impact on the diversity of current cotton genotypes, resulting in a reduction in genetic diversity. As a result, wild accessions became valuable pools of natural genetic diversity that can be used to expand cultivar genetic bases (Kirungu et al., 2018). Cotton production, on the other hand, is hampered by numerous biotic and abiotic stresses. Drought stress has emerged as the most serious threat to major cotton crop loss due to the global shortage of water (Ullah A. et al., 2017). Understanding the biochemical and genetic bases of cotton's drought response and developing drought-tolerant cotton varieties, is critical.

Several genes from wild resources improve tolerance to abiotic stress by indirectly detoxifying cellular reactive oxygen species (ROS) (Guo et al., 2017). Aldehyde dehydrogenase (ALDH) is a family of enzymes that catalyze the permanent conversion of aldehydes to acids to reduce the damage caused by abiotic stressors. Abiotic stress including drought, salinity, and high temperatures also cause a buildup of ROS which stimulates endogenous aldehyde formation via a lipid peroxidation chain reaction (Chen et al., 2015). Aldehydes and their detoxifying roles in plants are poorly understood. As a result, it is of immense importance to distinguish the approaches underlying the detoxification of aldehydes throughout abiotic stresses and to mine endogenous resistance genes (Guo et al., 2017). Thus, drought tolerance is complex and mutagenic. Therefore, an integrated transcriptomic, metabolomic, and functional analysis approach is required to make progress in drought tolerance (Oladosu et al., 2019). It is imperative to reveal this complex mechanism or to explore the relevant genes and pathways related to drought stress tolerance in cotton. Hence, understanding the molecular mechanisms and metabolic regulatory networks will help to improve the drought tolerance in cotton. Therefore, in the current study, integration of transcriptome and metabolome was performed at various time intervals in different plant parts to investigate the genetic and molecular networks and pathways underlying drought tolerance in three cotton semi-wild lines, namely, Marie-galantie85 (MG85) tolerant, Lattifolium40 (LT40) sensitive, and Upland cotton (CRI12) as a standard check. Virus induced gene silencing (VIGS) and overexpression-based functional characterization of candidate gene was also performed to validate its part in response to drought stress. The current study offers a comprehensive understanding and additional knowledge to our existing knowledge of both genetic and drought tolerance in cotton is mediated by molecular processes.

## Materials and Methods

### Planting Material, Plant Growth, and Drought Stress Treatment

Two semi-wild accessions of *G. hirsutum*, namely, Marie-galantie85 and Lattifolium40 that were originated from Mexico and distributed in Guadeloupe and Guatemala and a released variety CRI12 for drought tolerant, sensitive, and regular checks respectively were used for transcriptome and metabolome analysis. Marie-galantie85 was specifically used for functional validation of the genes associated with the key metabolites identified in this study through gene knockout, also referred to as Virus-Induced gene silencing. A variety of CRI12 was also used for drought tolerant, sensitive, and regular checks (Xu et al., 2020).

The seeds of these lines were sterilized and aseptically kept immersed in water for one day at 30°C before being placed for germination in an absorbent paper under 28°C of day and night temperature. They were placed in 16/8 h of alternating light and dark periods (Zhang et al., 2017). The experiment was set up in a greenhouse with three biological replications in a completely random design. The properly germinated seeds on paper rafts were transferred to a hydroponic setup of Hoagland solution (Hoagland and Arnon, 1950).

The VIGS and the wild-type seedlings were subjected to 17% PEG-6000 for PEG-induced drought at the three-leaf stage (Kirungu et al., 2020). PEG-6000 was used to simulate drought stress treatment in various crops. In *Triticum aestivum* L. (Faisal et al., 2019), in soybean (Basal et al., 2020), in tobacco (Yang et al., 2017), in *Stevia rebaudiana* (Ahmad et al., 2020), in Egyptian barley cultivars (Hellal et al., 2018), in peanut (Meher et al., 2018) and *Medicago sativa* L. (Zhang and Shi, 2018) used PEG-6000 induced treatment for drought stress.

The sampling parts of the materials were the roots and leaves. Sampling was done at 0, 24, and 48 h for transcriptome profiling sequence and 0, 3, 6, 9, 12, 24, and 48 for VIGS trials in three replications. A total of 54 samples were done for RNA-sequencing. The samples were then rapidly submerged in liquid nitrogen and kept at −80°C until RNA extraction was completed (Xu et al., 2020).

### Data Collection on Phenotypic, Physiological, and Biochemical Parameters

Phenotypic and physiological data including excised leaf water loss (ELWL), relative leaf water content (RLWC), chlorophyll content, and cell membrane stability (CMS) were recorded from three randomly sampled plants. We took leaf tissue samples for the determination of biochemical analysis. In the biochemical analysis, the antioxidant catalase (CAT), superoxide dismutase (SOD), hydrogen peroxide (H_2_O_2_), and malondialdehyde (MDA) enzyme activities were measured. All the antioxidants and oxidant enzymes CAT, SOD, H_2_O_2_, and MDA were evaluated using the Solarbio life sciences kit (www.solarbio.com) using the instructions of the manufacturer. For physiological traits, the following formulas were used to calculate the values.

ELWL = (FW – WW/DW) (Clarke and McCaig, 1982).
RLWC = (FW – DW)/ (SW – DW) × 100 (Barrs and Weatherley, 1962).
CMS = [(1 – T1/T2)/(1 – C1/C2)] × 100 (Blum and Ebercon, 1981).

Note, FW = Fresh weight, SW = Saturated weight, WW = Wilted weight, DW = Dry weight, T1 = Reading 1, T2 = Reading 2, C1 = Control 1, C2 = Control 2.

### cDNA Library Preparation, and RNA Sequencing

To isolate RNA samples from both leaf and root tissues for RNA-Seq analysis, Trizol R reagent (Invitrogen, Waltham, MA, USA) was used. Using an agarose gel electrophoresis and a NanoDrop 2000 spectrophotometer, the quality and concentration of RNA were assessed (Thermo Fisher Scientific, USA) (Yu et al., 2017). The Agilent 2100 Bioanalyzer RNA Nanochip was used to complete a more precise RNA quantification (Agilent Technologies, Waldbronn, Germany) (Shi et al., 2018). The cDNA fragments were then cleaned using a Qia Quick PCR extraction kit that was corrected at the ends with added poly(A) and ligated to Illumina sequencing adapters. Gene Denovo Biotechnology Co. used the Illumina HiSeqTM 2500 to amplify and sequence PCR products. The size of the ligation products was determined using agarose gel electrophoresis from Gene Denovo Biotechnology Co (Guangzhou, China). PCR amplified and sequenced the PCR using the Illumina HiSeqTM 2500. Tophat2 (V.2.0.13) was used to extract clean reads from raw reads, which were then associated to the reference genome of *G. hirsutum* (http://cottonfgd.org) to generate mapped reads (Kim et al., 2013). Cufflinks v2.2.1 was used to analyze gene expression and to calculate the differences between the treatment and control fragments per kilobase of transcript per million fragments (FPKM) values. Readings from the transcriptome were measured and adjusted to fragments per kilobase of transcript per million fragments linked to the reference genome (Trapnell et al., 2014).

### DEGs Identification

R software (http://www.r-project.org/) was used to find differentially expressed genes between samples. The false discovery rate (FDR) and log2 Fold Change thresholds for differentially expressed genes (DEGs) were set at < .05 and > 1, respectively. The DEGs were then submitted to a GO function and KEGG pathway enrichment analysis. First, through the AgriGO web tool (http://systemsbiology.cau.edu.cn/agriGOv2/), all DEGs were mapped to GO (Gene and Consortium, 2000). Pathway enrichment analysis revealed considerably enhanced metabolic transduction pathways when DEGs were compared to the entire genomic background.

### WGCNA Analysis

To transfer genes into co-expressed modules, weighted gene co-expression network analysis (WGCNA) was done in the R package (Zhang and Horvath, 2005; Langfelder and Horvath, 2008). Before generating an adjacency matrix, the FPKM values were normalized. WGCNA software was used to import the phenotypic data, and a correlation-based relationship between accessions, time points, and gene modules was done using the default settings. The WCGNA software was used to transform the adjacency matrix into TOM (topological overlap matrix). Transcripts with comparable expression patterns were clustered into one module once the network was created, and eigengenes were determined for these modules. Using Cytoscape's default parameters, each module's genes were exported.

### Co-expression Network and Phylogenetic Analysis of Key Genes

Leaf and root tissues of the three semi-wild cotton species Marie-galantie85, Lattifolium40, and Upland cotton at 0-, 24-, and 48-h time points were used as phenotype data in the WGCNA analysis. Thirteen unique gene modules were recognized from RNA Seq data by entering WGCNA using the FPKM values. Coexpression network analysis of the three highly and positively correlated to drought modules namely brown, yellow, and pink was established by Cytoscape v.3.7.2 (Shannon et al., 2003). Cotton functional genomics database website (www.cottonfgd.org/) was used to download the protein sequence of the candidate gene for *G. hirsutum and G. arboreum*, while *G.raimondii, A. thaliana, Theobroma cacao, Brassica rapa, Glycine max, Medicago truncatula, Oryza sativa, Triticum aestivum, Zea mays*, and *Sorghum bicolor* were downloaded from phytozome (https://phytozome.jgi.doe.gov). The ClustalX tool was used to align the full-length ALDH protein sequences (Larkin et al., 2007) and MEGA 7 was used to create the phylogenetic tree using the neighbor-joining method with 1000 bootstrap replications (Tamura et al., 2011).

### Metabolite Profiling, and Data Analysis

The tender leaves and roots of cotton at the three-leaf seedling stage were used for metabolite profiling by the liquid chromatography-mass spectrometry (LC-MS) approach. To detect reproducibility under the same treatment, the sample extracts were mixed to prepare a quality control sample (QC). During the LC-MS analysis, QC samples are arranged by mixing sample extracts to analyze the recurrence ability of samples under the same processing method. In the process of instrument analysis, a QC sample is usually inserted into every 10 test analysis samples to investigate the repeatability of the analysis process. Three biological replications were maintained in sampling and metabolome analysis (Zhou et al., 2012).

### Cloning and Transformation of *Gh_A06G1257* in *A. thaliana*

RNA sequencing of *G. hirsutum* yielded a substantially upregulated gene, which was then transformed into *Arabidopsis thaliana* (Colombia-0). The PCR study with forward (CGGCCATTTAAATAGTGGATTCGG) and reverse (GCCACCATCTATCCTCAACGA) pair of primer sequences of *Gh_A06G1257* synthesized from Invitrogen, Beijing, China, verified the pWM101-35S: *Gh_A06G1257* build in *Agrobacterium tumefaciens* GV3101. The floral dip approach was used to transform wild-type *A. thaliana* plants.

The infiltration media was prepared as prescribed previously (Lu et al., 2019). The seedlings were moved to a growing environment with a temperature of 25°C and a 16 light/8 dark hours cycle from the selection medium at three-leaf stages. The little plastic pots were filled with a 1:1 mix of vermiculite and humus (Sadau et al., 2021).

The first-generation seeds were collected after the seedlings from generation T0 had been grown to set seeds (T1). True lines were found by estimating the antibiotic-selectable marker segregation ratio of 3:1 after T1 seeds were sown in antibiotic media. Only the lines with a 100% success rate were chosen for T3 generation growth. After RT-qPCR, T3 homozygous lines were chosen from a T2 generation. Three of the six ALDH transgenic lines that were successfully transformed (OE-1, OE-9, and OE-10) were chosen from a T2 generation. The RT-qPCR prototype was *Gh_A06G1257* forward primer sequence and *Gh_A06G1257* reverse primer sequence with complete complementary DNA (cDNA). T3 homozygous generation was used to conduct the phenotypic studies.

### Determination of Drought Tolerance in the Transgenic Lines

The plants were subjected to drought stress treatments after 21 days of growth. Samples from leaves were collected from transgenic lines and control. The pots were then watered with water containing 15% PEG-6000 treatment was used to alleviate drought tension. After 8 days, physiological and phenotypic traits were observed. All measurements were replicated three times biologically and three times technically (Li et al., 2019).

### Germination Rate and Root Elongation Determination

Drought simulated stress conditions were used to assess the germination percentage and root length of transgenic lines and wild form. OE-1, OE-9, OE-10, and wild-type seeds were sown in.5 MS plates complemented with 0, 100, 200, and 300 mM mannitol concentrations to simulate drought. After 10 days, the germination rate was assessed. Transgenic and wild-type seeds were seeded for 6 days in 0.5 MS media before being moved to 0.5 MS supplemented with different amounts of 0, 100, 200, and 300 mM mannitol for the root length assay. All measurements were replicated three times biologically and three times technically (Li et al., 2019).

### Drought Stress Treatment for VIGS in Cotton

The functional characterization of substantially upregulated gene expression in *G. hirsutum* was investigated using this approach. A gene *Gh_A06G1257* with 296 bp fragments was transformed by Forward (CTGTGAGTAAGGTTACCGAATTCTCTAGAGAGATGTGGAATCCCCTTGGAA) and Reverse (TCGAGACGCGTGAGCTCGGTACCGGATCCACCTTCGAACTCCCCGTGA) primer sequences into pTRV vector using enzymes XbaI and BamHI to develop a 35S promoter-driven pTRV2: *ALDH7B4*. The promoter cells of *A. tumefaciens* LBA4404 were deformed with the recombinant vector by freezing and thawing (Velásquez et al., 2009). Wild, pTRV: 00 were used positively with Phytoene desaturase (PDS) as a negative control.

### RT-qPCR Analysis

To validate RNA-Seq data, DEGs were verified by RT-qPCR using (Schmittgen and Livak, 2008) procedures. Primers were created using NCBI (https://www.ncbi.nlm.nih.gov) and gene sequences from *G. hirsutum*. Quantitative PCR was performed using an SYBR® Green PCR Master Mix Kit (Applied Biosystems, Foster City, CA, USA) and an ABI-7900 system. The 2-^ΔΔC^T was used to determine the relative gene expression (Schmittgen and Livak, 2008). Internally, *GhActin* was employed as a reference. The RT-qPCR experiment was carried out in three biological and technical replications (Chen et al., 2015).

### Statistical Analysis

Chenomx NMR Suite 7.7 was used to do the multivariate statistical analysis, principal component analysis (PCA), partial least squares determinant analysis (PLS-DA), and orthogonal projections to latent structures (OPLS-DA). The variance in the data matrix was presented using PCA. PLS-DA can maximize the differentiation between groups, which helps find differential metabolites. OPLS-DA data was used to examine subsequent model tests and differential metabolite screening. Fisher's exact test in R was used to calculate statistical significance. The Benjamini-Hochberg correction was used to adjust the false recovery rate (FDR) in the transcriptome analysis (Noble, 2009). Coexpression network analysis was established by Cytoscape v.3.7.2 (Shannon et al., 2003). The SPSS program was used to perform both t-tests and ANOVA. Significant differences were declared at the *p* < 0.05 probability level (Worley and Powers, 2015).

## Results

### Phenotypical and Biochemical Response of Cotton Lines Under Drought Stress

**Figure 1:**
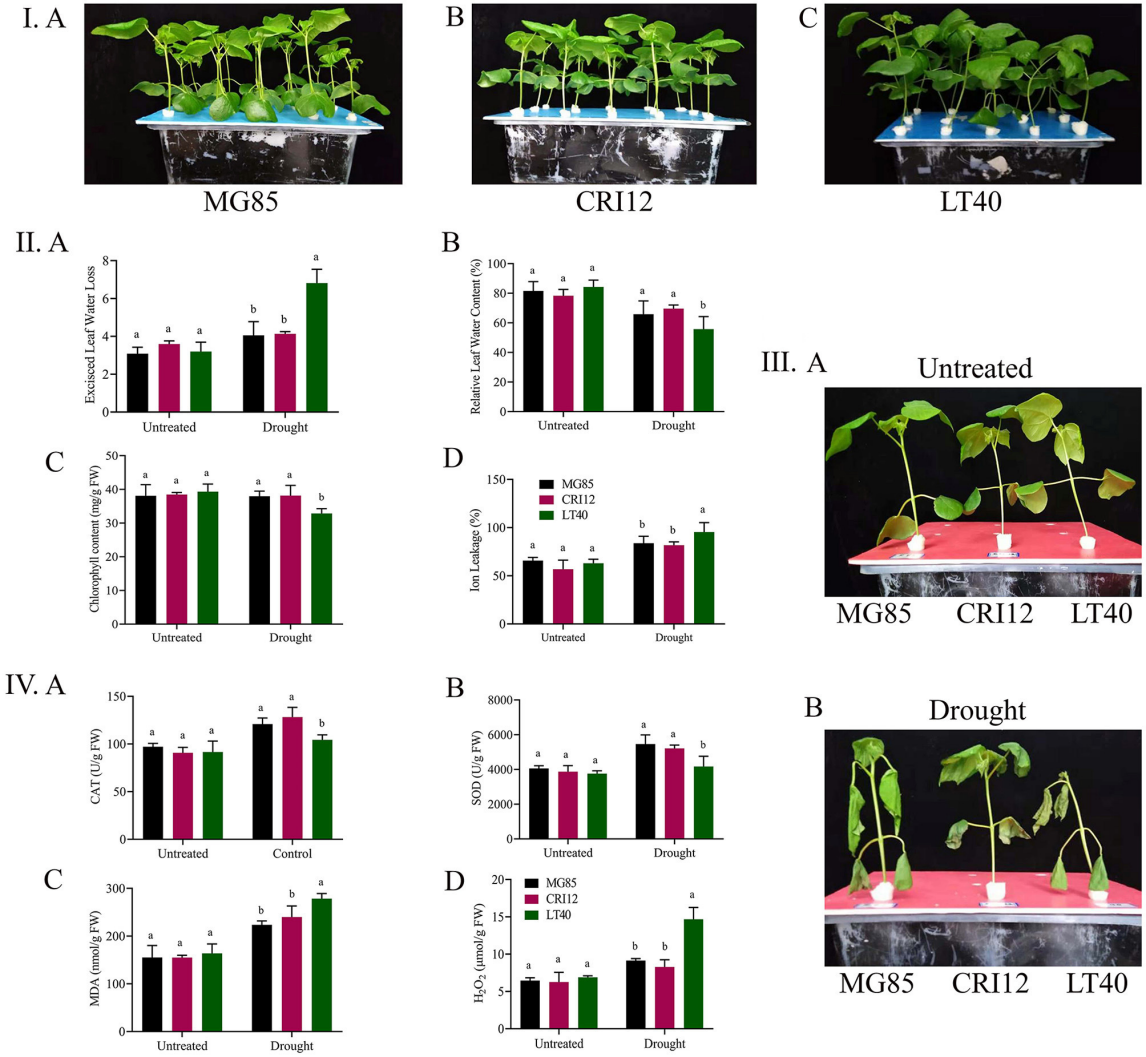
Cotton physiological and biochemical parameters determination. **(I)** A Marie-galantie85, B Upland cotton, C Lattifolium40. **(II)** Phenotype parameters evaluation, A Excised leaf water loss, B Relative leaf water content, C Chlorophyll content, D Cell membrane stability. **(III)** A Representative images of Marie-galantie85, Upland cotton, and Lattifolium40 before treatment B Representative images of (Marie-galantie85, Upland cotton, and Lattifolium40) after Drought treatment. **(IV)** A Catalase, B Super oxidase, C Malondehyde, D Hydrogen peroxide. SE is reflected by error bars. Using the Least Significance Difference (LSD) means on the same graph tracked by the same letter are not statistically different at the 5% probability level, Drought: supplementing with 17% PEG-6000 solution.

Wilting was observed in the plants under drought stress with reduced evaporation. There were no obvious differences in the phenotype of three semi-wild cotton lines in response to drought stress (Figure 1I). Clear and significant differences (*p* < 0.05) were recorded in the case of physiological and biochemical parameters (Figure 1II). Chlorophyll and relative water contents in MG85 and CRI12 were higher as compared to LT40. A lower ion leakage with a higher excised leaf water loss was observed in LT40 as compared to CRI12 and MG85 (Figure 1III).

Higher activities of CAT and SOD were observed in MG85 and CRI12 under drought stress conditions, however lower CAT and SOD were observed in the case of LT40. Moreover, we also measured the H_2_O_2_ and MDA activities. We found out that they were higher in LT40 as compared to MG85 and CRI12. Thus, suggesting that MG85 and CRI12 were more resistant to drought as compared to LT40 (Figure 1IV).

### Transcriptome Profiling of Semi-wild Cotton Accessions

We selected semi-wild cotton lines, namely, Marie-galantie85, Lattifolium40, and Upland cotton for the transcriptome sequencing in response to drought treatment. After sequencing, 54 transcriptome libraries were obtained. For the accuracy of the subsequent analysis of the new data, reads with lower, linker sequences, sequences having >10% unknown N were deleted. After filtering the new data, we finally acquired high-quality clean reads of 2.66 Gigabytes in all the 54 libraries (Table 1). The average length of each clean-read was 150 bp. We, therefore, acquired over 95% high-quality clean reads. The Q20 and Q30 base percentages were all above 96 and 90%, respectively, and the GC content was higher than 43% (Table 1). Bowtie2 was used for the screening of high-quality reads and then comparing them with *G. hirsutum* reference genome by TopHat2 software. The samples were tested for Pairwise repeatability and the correlation coefficient was all above 84%, indicating that the samples were reproducible.

**Table 1:** A summary of current transcriptome results.

**Sample**	**Raw reads**	**Clean reads**	**Total mapped reads**	**Unique mapped reads**	**GC (%)**	**Q20 (%)**	**Q30 (%)**
TRL0-1	48,382,870	47,225,346	46,864,548	38,448,228	47.99	97.61	93.23
TRL0-2	52,579,728	51,266,772	51,111,992	43,772,867	47.24	97.55	93.10
TRL0-3	56,336,308	55,247,256	55,065,692	47,160,405	46.70	97.88	93.91
TGL0-1	62,664,822	61,409,970	61,189,338	52,802,603	47.22	97.86	93.87
TGL0-2	52,842,324	51,850,414	51,726,288	44,713,330	46.71	97.86	93.86
TGL0-3	58,919,954	57,594,852	57,416,494	49,525,417	47.14	97.61	93.28
TSL0-1	58,559,786	57,179,842	57,016,934	48,485,182	46.99	97.63	93.34
TSL0-2	59,147,410	57,876,556	57,665,152	49,235,289	47.29	97.73	93.55
TSL0-3	56,103,088	55,022,828	54,945,274	47,130,318	45.29	97.85	93.88
TRR0-1	59,661,212	58,348,918	58,250,544	47,177,073	46.50	97.65	93.41
TRR0-2	60,836,394	59,464,474	59,424,308	47,696,256	46.18	97.69	93.52
TRR0-3	60,472,180	59,089,838	59,049,864	48,394,946	46.05	97.67	93.45
TGR0-1	53,556,182	52,473,026	52,431,124	43,410,768	46.36	97.72	93.55
TGR0-2	52,873,460	51,652,388	51,628,582	41,462,144	46.52	97.64	93.38
TGR0-3	53,740,848	52,592,468	52,555,948	43,484,717	46.13	97.72	93.57
TSR0-1	35,554,402	34,230,188	34,195,512	27,253,893	46.55	96.47	90.37
TSR0-2	37,148,188	35,631,744	35,578,158	27,782,277	46.26	96.37	90.17
TSR0-3	36,465,000	35,138,014	35,100,842	28,224,201	46.08	96.49	90.41
TRL24-1	38,588,574	37,466,818	37,364,512	31,856,229	44.36	96.86	91.27
TRL24-2	43,266,850	41,820,656	41,748,212	35,299,949	44.34	96.70	90.92
TRL24-3	37,506,180	36,261,436	36,195,632	30,764,724	43.88	96.68	90.89
TGL24-1	42,139,862	40,793,736	40,723,212	34,645,495	43.91	96.75	91.05
TGL24-2	38,490,510	37,387,542	37,280,882	31,901,237	43.62	96.90	91.40
TGL24-3	51,608,270	50,429,954	50,380,624	43,125,825	44.35	97.49	92.92
TSL24-1	37,963,532	36,924,042	36,842,624	31,105,640	44.37	96.94	91.47
TSL24-2	40,970,448	39,753,298	39,526,760	33,257,300	45.50	96.88	91.29
TSL24-3	32,655,298	31,682,966	31,612,760	26,842,030	45.94	96.82	91.11
TRR24-1	36,393,502	35,239,978	35,227,504	29,516,296	45.80	96.74	90.96
TRR24-2	39,448,514	38,355,908	38,333,978	32,361,413	45.27	96.91	91.37
TRR24-3	44,130,164	42,809,914	42,760,620	35,729,344	46.21	96.80	91.09
TGR24-1	39,080,012	37,735,156	37,659,594	31,708,405	46.00	96.61	90.64
TGR24-2	60,082,788	59,164,730	59,100,514	50,716,629	44.57	98.36	95.27
TGR24-3	62,648,430	61,644,858	61,545,300	52,233,829	44.36	98.35	95.25
TSR24-1	53,922,604	53,047,176	53,008,684	44,163,965	45.88	98.30	95.10
TSR24-2	59,216,670	58,302,694	58,251,088	48,640,679	45.05	98.33	95.21
TSR24-3	61,298,808	60,309,438	60,271,016	50,511,717	45.15	98.28	95.08
TRL48-1	57,665,814	56,787,410	56,704,326	48,914,483	44.74	98.40	95.40
TRL48-2	59,600,270	58,630,866	58,514,704	50,438,767	44.42	98.31	95.15
TRL48-3	62,355,000	61,249,494	61,063,896	52,450,181	45.68	98.21	94.90
TGL48-1	50,097,144	49,265,584	49,029,832	42,334,742	45.24	98.26	95.03
TGL48-2	51,026,702	48,797,200	48,707,336	41,259,576	45.82	96.67	91.04
TGL48-3	49,785,362	48,446,686	48,357,420	41,420,251	45.16	97.28	92.39
TSL48-1	44,610,152	43,327,706	43,165,214	36,405,082	45.90	97.23	92.27
TSL48-2	45,646,602	44,351,926	44,240,168	37,396,188	44.09	97.20	92.27
TSL48-3	45,866,752	44,708,516	44,645,818	38,058,545	44.35	97.32	92.53
TRR48-1	50,231,244	48,964,774	48,922,846	41,803,375	44.44	97.31	92.50
TRR48-2	39,102,736	38,217,636	38,189,310	32,714,043	44.18	97.43	92.78
TRR48-3	45,484,738	44,342,814	44,311,208	37,838,745	44.25	97.35	92.59
TGR48-1	51,054,758	49,757,474	49,700,324	41,707,916	43.79	97.34	92.59
TGR48-2	66,423,520	65,320,396	65,236,086	55,097,529	43.45	98.32	95.22
TGR48-3	60,719,100	59,806,544	59,777,658	49,964,718	43.64	98.36	95.30
TSR48-1	51,940,624	51,138,914	51,064,996	43,096,431	44.34	98.32	95.19
TSR48-2	54,843,004	53,977,846	53,945,150	45,435,202	44.13	98.35	95.26
TSR48-3	62,110,754	61,093,238	60,981,072	52,012,735	44.66	98.25	95.01
Total	2,723,819,448	2,660,610,218	2,655,607,474	2,238,889,129	–	–	–

*TRL-0, leaf tissue of Marie-galantie85 at 0 h; TRR-0, root tissue of Marie-galantie85 at 0 h; TGL-0, leaf tissue of Upland cotton at 0 h; TGR-0, root tissue of Upland cotton at 0 h; TSL-0, leaf tissue of Lattifolium40 at 0 h; TSR-0, root tissue of Lattifolium40 at 0 h; TRL-24, leaf tissue Marie-galantie85 at 24 h; TRR-24, root tissue Marie-galantie85 at 24 h; TGL-24, leaf tissue of Upland cotton at 24 h; TGR-24, root tissue of Upland cotton at 24 h; TSL-24, leaf samples of Lattifolium40 at 24 h; TSR-24, Lattifolium40, root samples at 24 h; TRL-48, leaf samples of Marie-galantie85 at 48 h; TRR-48, root tissue Marie-galantie85 at 48 h; TGL-48, leaf samples of Upland cotton at 48 h; TGR-48, root tissue of Upland cotton at 48 h; TSL-48: Leaf tissue of Lattifolium40 at 48 h; TSR-48, root samples of Lattifolium40 at 48 h; 1, 2, and 3 stands for three replications*.

### DEGs Identification and Drought Stress Treatment

**Figure 2:**
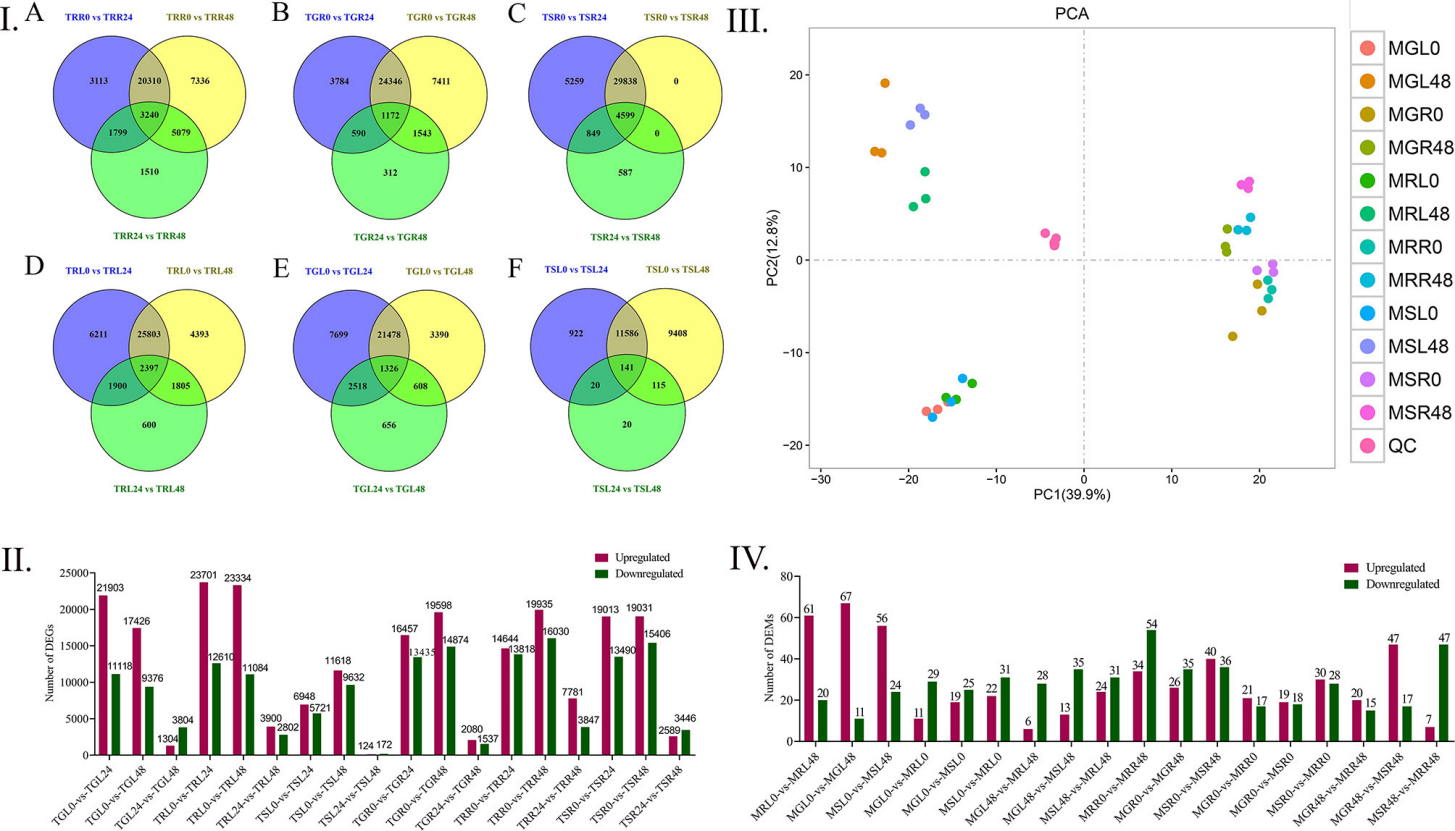
Distribution of DEGs and DEMs in different time points of *Gossypium* accessions. **(I)** Gene distributions among different materials, A Differential gene distribution among different comparison groups of MG85 leaves. B Differential distribution of LT40 gene among different comparison groups. C Gene distribution among different comparison groups of CRI12 leaves, D MG85 differences between different comparison groups Gene distribution, E LT40 differential gene distribution among different comparison groups, F CRI12 differential gene distribution among different comparison groups. **(II)** Number of DEGs identified by pairwise comparisons between cotton lines and drought treatments. Upregulated (Red) and downregulated genes were quantified. Based on |log2 (fold change) > 1| and *p* < 0.05 were considered as significantly differential genes. **(III)** Principal Component Analysis Model, where MRL, Marie- galantie85 leaves; MRR, Marie-galantie85 Root; MGL, upland cotton leaf; MGR, upland cotton root; MSL, Lattifolium40 leaf; MSR, Lattifolium40 Root. **(IV)** Differentially Expressed Metabolites, upregulated (Red) and downregulated genes were quantified. LC-MS metabolite analyses were used to detect the metabolites from leaf and root tissues. DEGS, differentially expressed genes; DEMs, differentially expressed metabolites; LC-MS, Liquid chromatography-Mass spectrometry.

We found that the upregulated genes were higher in number as compared to the downregulated genes in both the leaves and roots. Specifically, TRL0 vs. TRL24 (23701 vs. 12610), TGL0 vs. TGL24 (21903 vs. 1118), and TSL0 vs. TSL48 (11618 vs. 9632) in leaves. Whereas TRR0 vs. TRR48 (19935 vs. 16030), TGR0 vs. TGR48 (19598 vs. 14874), and TSR0 vs. TSR48 (19031 vs. 13490) were highly upregulated and downregulated genes in roots (Figure 2II).

The reason for higher DGEs number in roots at different time points of drought application could be that they are the first to be in contact with PEG-6000, thus triggering the drought response. The amount of differently expressed genes in root tissues grew over time, owing to the roots' direct interaction with PEG-6000, which resulted in more sustained drought response in the roots. Intersection and union analysis of differentially expressed genes at different time points in the same material was performed by applying Venny online software (Figure 2I).

### Analysis of Differential Gene Expression Trends

The expression patterns of genes change with conditions such as specific environments and time. The trend analysis of genes that were differentially expressed was performed by STEM software for the expression of leaf and root regulatory genes at different time points. The differentially expressed genes of the experimental materials were divided into 8 gene expression trends. According to the standard of (*p* < 0.05), the gene expression trend showed the same significant trend (trend 1, trend 6, and trend 7) with time in the three materials in both leaves and roots (Table 2).

**Table 2:** Trends of DEGs expressions levels in MG85, LT40, and CRI12.

	**Root**				**Leaf**			
	**Trend 0**	**Trend 1**	**Trend 6**	**Trend 7**	**Trend 0**	**Trend 1**	**Trend 6**	**Trend 7**
MG85	6,512	8,437	11,834	6,109	6,512	9,412	20,725	3,723
LT40	7,167	7,678	16,047	4,561	7,167	4,993	9,369	2,533
CRI12	4,817	10,066	14,708	5,162	4,817	8,911	18,032	2,550

*MG85, marie-galantie85; LT40, Lattifolium40; CRI12, upland cotton*.

### Analysis of Metabolites in Cotton Semi-wild Lines Under Drought Stress

According to the principal component analysis of the six quality control samples gathered together, the dispersion is very small, indicating that the stability of the instrument is better in the analysis of metabolites, and the results of metabolite analysis are reliable. Thirty-six samples were divided into four components by the main components PCA1 (39.9%) and PCA2 (12.8%). The main component PCA1 (39.9%) separated the leaves and root samples, indicating that under drought stress, leaves and roots respond differently (Figure 2III). The main component PCA2 (12.8%) separates the samples at different treatment time points of the material, and the difference in the roots is small. It is indicated that the changes in leaves are more obvious at different treatment time points of drought stress. This may be due to the sensitivity of leaf tissues to stress as compared to root tissues. LC-MS analysis was helpful to identify 445 metabolites totally (Supplementary Table 4). According to the analysis of the differentially expressed metabolites, the highest upregulated metabolites were recorded in the leaves at MGL0 vs. MGL48 (67 metabolites), MRL0 vs. MRL48 (61 metabolites), and MSL0 vs. MSL48 (56 metabolites), respectively, whereas the highest downregulation of metabolites was found in roots from MRR0 vs. MRR48 (54 metabolites), MSR0 vs. MRR48 (47 metabolites) and MSR0 vs. MSR48 (36 metabolites) successively (Figure 2IV).

### GO Function Enrichment Analysis

The identified differentially expressed genes in both the roots and leaves tissues were mainly enriched in the molecular functions, cell components, and the biological processes of functional categories (Supplementary Figure 1). In MG85, the leaf tissues were enriched in biological and molecular functions, whereas the root tissues were enriched only in biological processes. In CRI12, both tissues are enriched in two functions, namely, biological and molecular, and biological and cellular, respectively. Differently from the above lines LT40 enriched in all the three GO functions biological, cellular, and molecular in leaf tissues only, there is no significant enrichment in the root tissues (Supplementary Figure 1). In the biological process, the most important DEGs were involved in metabolic processes (GO:0008152), cellular processes (GO:0009987), biogenesis (GO:0071840), and single organism processes (GO:0044699), etc. On the other hand, in cellular components, DEGs were mainly involved in cells (GO:0005623), cell parts (GO:0044464), organelles (GO:0043226), macromolecular complex (GO:0032991), and extracellular region (GO:0005576). The molecular functions mainly included binding (GO:0005488) and catalytic activity (GO:0003824). Among them, we found that most of the DEGs were enriched in the metabolic processes in the tissues of different materials. Thus revealing that primary metabolites and secondary metabolites play an essential part in response to drought stress.

### WGCNA Analysis for the Identification of Hub Genes

**Figure 3:**
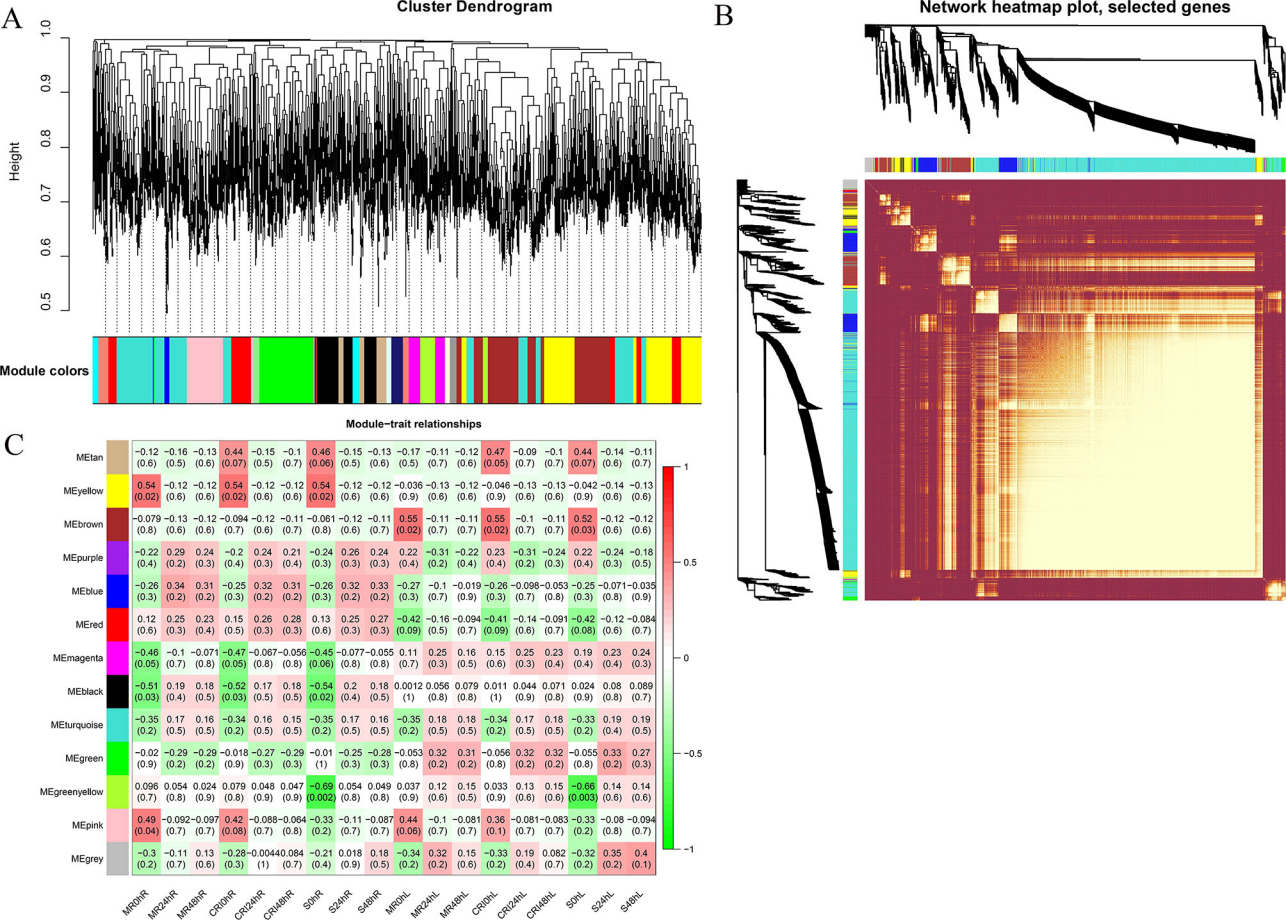
WGCNA revealed gene networks and major candidate genes for drought **(A)** Network building of three upland cotton accessions using cluster dendrograms. **(B)** Heatmap of the network of genes that were subjected to the coexpression module calculation. **(C)** Pearson correlation-based module-trait relationships. From green to red, the color key symbolizes *r*^2^ values ranging from −1 to 1. Because of the highest weight inside the module, each network's hub genes are indicated in red and gene descriptions are coded using annotations. WGCNA, Weighted gene co-expression network analysis.

Here we used the FPKM values of commonly expressed DEGs to perform a weighted gene coexpression network analysis for the identification of hub genes associated with drought stress tolerance. A cluster dendograms was generated to see the number of modules along with DEGs, a network heatmap was also generated from the genes located in each of the identified module (Figures 3A,B). Using WGCNA, thirteen different modules were detected. Out of these thirteen modules, three modules (Yellow, brown, and pink) were found to be highly and positively associated with our phenotype. The yellow module contains 1,568 genes, the brown module contains 1,234 genes whereas 765 genes were present in the pink module. The yellow module has significant associations with MG85 0 h, CRI12 0 h, and LT40 0 h in root with *r* = 0.02 and *p* = 0.54, respectively. Brown modules have significant correlations with MG85 0 h, CRI12 0 h, and LT40 0 h in leaf with *p* = 0.02, *p* = 0.02 and *p* = 0.03 with *r*^2^ values of 0.55, 0.55, and 0.52, successively. Pink modules have significant associations with MG85 0 h and CRI12 0 h with *r*^2^ = 0.42, 0.44 and *p* = 0.49 and 0.42, respectively (Figure 3C). Cytoscape v.3.7.2 was used for the network visualization.

### Network Visualization, RT-qPCR Validations, and Phylogenetic Analysis for Key Genes Linked to Drought Stress

**Figure 4:**
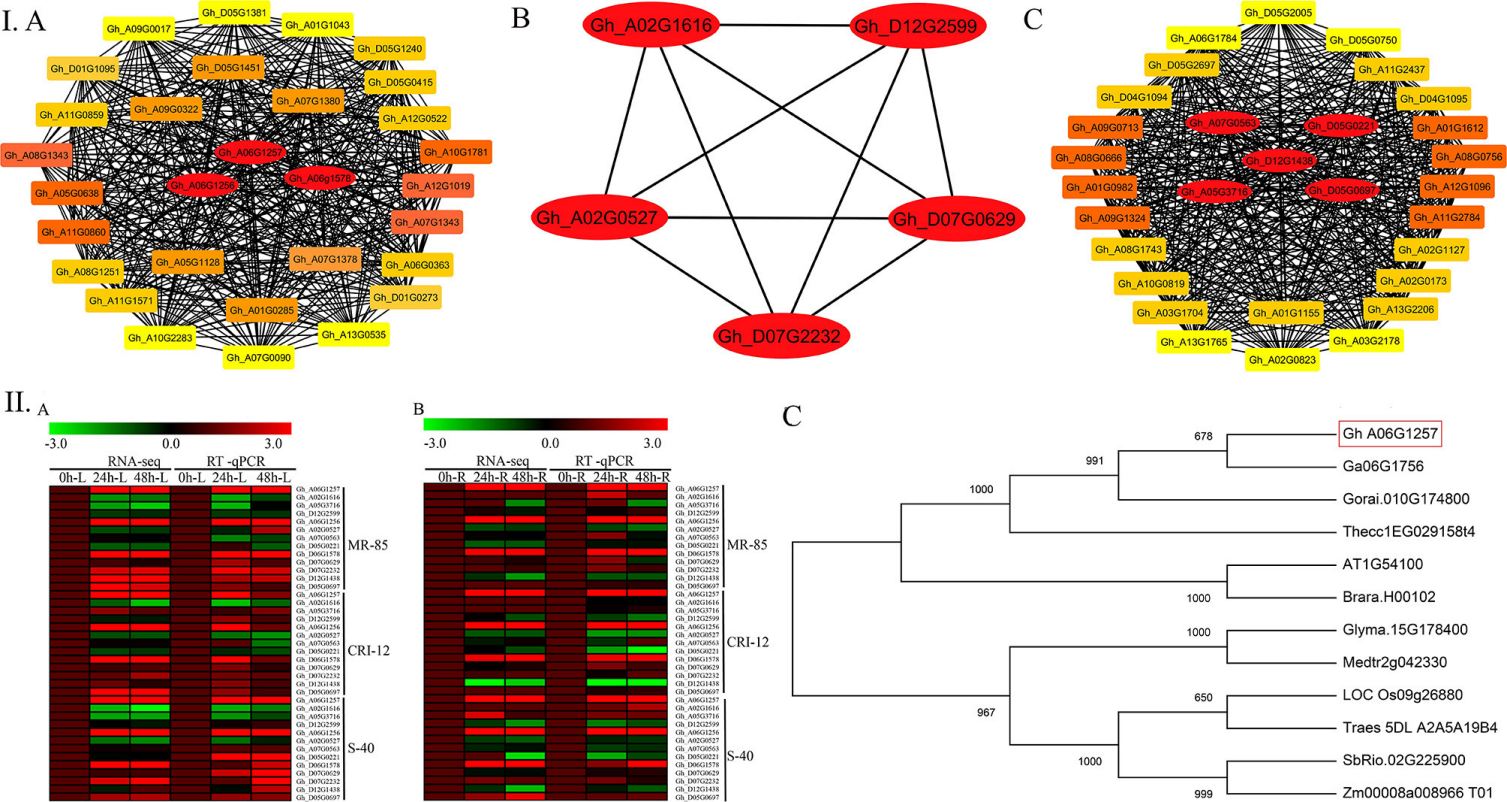
Hub genes identification for drought stress tolerance and RT-qPCR analysis. **(I)** A Brown module, B Pink module, C Yellow module. **(II)** Gene expression in the cotton accessions is represented as a heat map. A Heat map of RNA Seq (FPKM) and RT-qPCR of leaf tissues in log2 fold change. B Heat map of RNA Seq (FPKM) and RT-qPCR (RT-qPCR) of root tissues in log2 fold change. C Phylogenetic tree analysis for homologous genes. FPKM, Fragments per kilobase of transcript per million fragments; RT-qPCR, Real time quantitative polymerase chain reaction.

WGCNA helped us to import the top 30 genes for each of the significant modules to build the coexpression networks. The gene lists were further used for the network visualizations. We used Cytoscape software for this purpose. To identify the hub genes, a built-in Cytoscape extension by the name of “cytohubba” was used (Shannon et al., 2003). Five hub genes from the yellow module include *Gh_A07G0563, Gh_D05G0221, Gh_A05G3716, Gh_D12G1438*, and *Gh_D05G0697*, the brown module contains three hub genes (*Gh_A06G1257, Gh_A06G1256*, and *Gh_D06G1578*), and the pink module has five hub genes, namely, *Gh_A02G1616, Gh_D12G2599, Gh_D07G2232, Gh_A02G0527*, and *Gh_D07G0629*. The most interesting thing is that all the thirteen hub genes belong to the same ALDH family. Furthermore, the expression profiles of all hub genes in different plant parts under drought stress were validated via RT-qPCR. Results from RT-qPCR suggest that the *Gh_A06G1257* gene, having the highest expression under drought stress, might be the true candidate responsible for drought stress tolerance. We further validate this gene on a functional basis (Figure 4).

Two distinct clusters were formed as a result of phylogenetic analysis. The candidate gene *Gh_A06G1257* together with the two Gossypium species together with *A. thaliana, T. cacao*, and *B. rapa* were grouped in one cluster. *G. max, M. truncatula, O. sativa, T. aestivum, Z. mays*, and *S. bicolo*r were also grouped in the second cluster.

### Overexpression of *Gh_A06G1257* in Arabidopsis Increases Tolerance to Drought Stress

**Figure 5:**
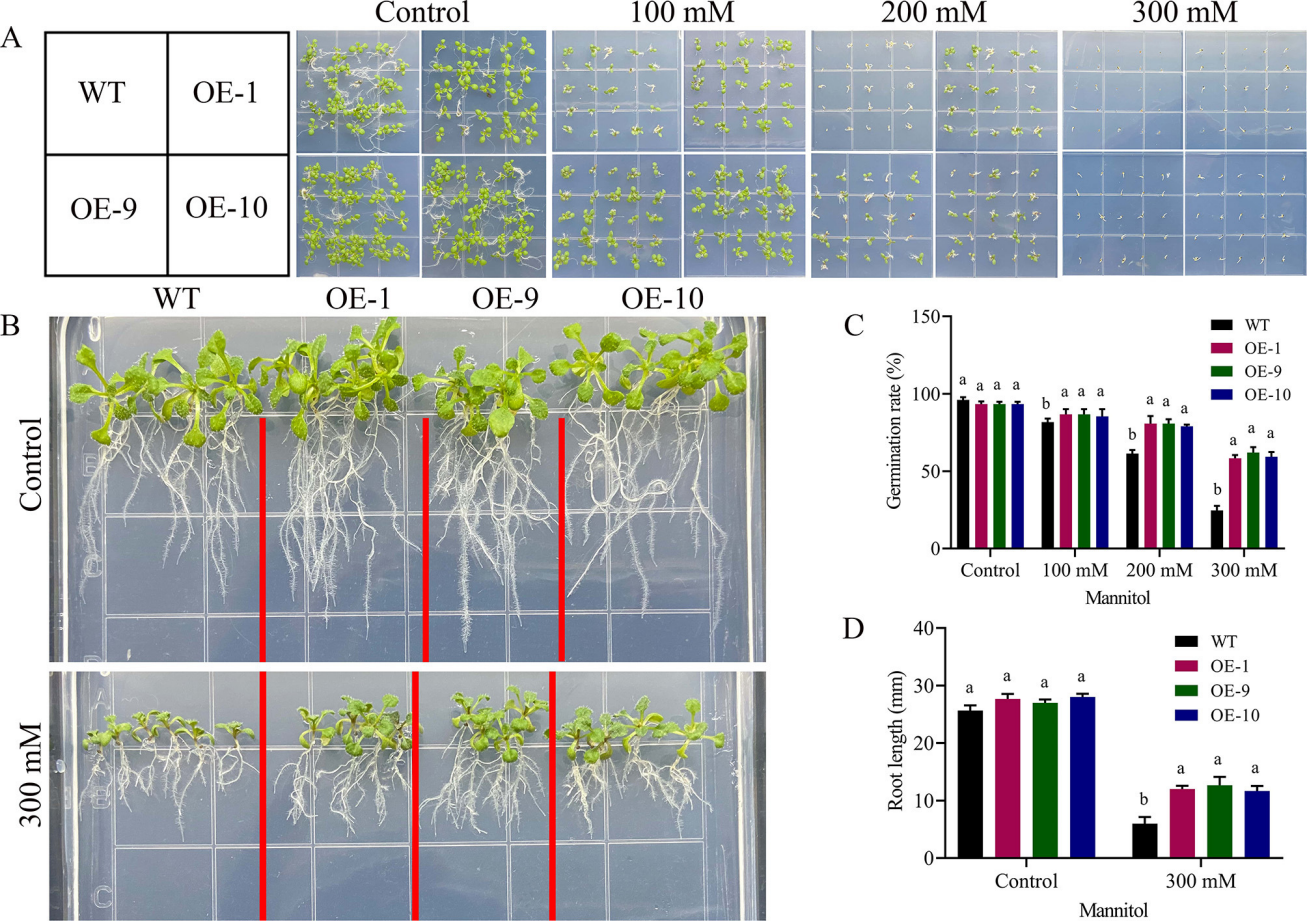
Germination and root length determination **(A)** Arabidopsis has grown on MS medium with 100 mM, 200 mM, and 300 mM mannitol evaluation. **(B)** Root length determination after drought treatment **(C)** Germination assays of Arabidopsis growth in different concentrations of mannitol, **(D)** Root length determination of Arabidopsis growth in 0- and 300-mM concentrations of mannitol. The error bars reflect the standard error. Using the LSD, means on the same graph, with the similar letter are not significantly different at the 5% probability level. LSD, the least significant difference. OE, overexpressed; WT, wild-type; mM; millimole.

We performed PCR and RT-qPCR analysis to see the expression level of six positive lines. OE-1, OE-9, and OE-10 overexpressed lines conformed with the highest expression level (**Figure 8I**). Seedlings of overexpressed lines were exposed to drought for studying the functions of *Gh_A06G1257* during drought stress. The wild-type and transgenic seedlings grew and germinated uniformly in the MS medium. On the other hand, the MS medium containing 300 mM mannitol seedlings of OE lines (OE-1, OE-9, and OE-10) showed better germination, grew faster than wild-type seedlings, and demonstrated a drought-resistant phenotype. Increased mannitol concentration resulted in a steady decrease in the rate of germination. In normal conditions, germination was calculated to be >90% in both wild and transgenic lines Figures 5A,B. However, under drought stress treatments, wild-type seedlings showed a decrease in germination rate to <25%, while a significantly higher germination rate of 60% was observed in transgenic lines in 300 mM mannitol. Under mannitol treatment, longer roots (13 mm) were observed in the lines with over-expressed ALDH gene while in wildtype a shorter root length of 6 mm, suggesting overexpressed lines are more resistant to drought stress (Figures 5C,D).

### Physiological Parameters Evaluation in Arabidopsis Under Drought Stress

**Figure 6:**
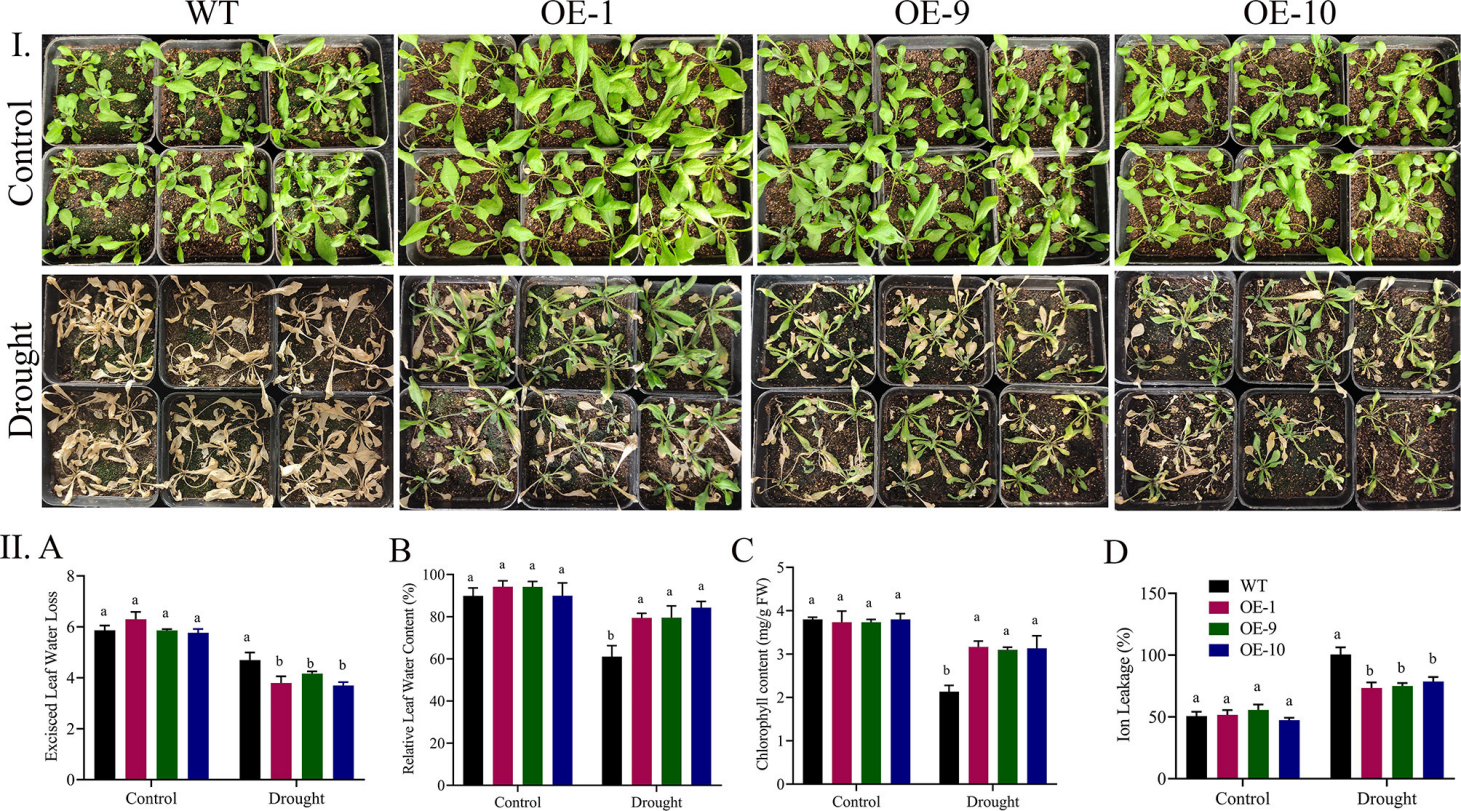
**(I)** Physiological parameters evaluation in ALDH overexpressed lines under drought stress. **(II)** A Transgenic lines and wild type under control and treatment. A Relative leaf water content, B excised leaf water loss, C chlorophyll content, and D Ion leakage after drought stress treatment. The error bars reflect the SE. Significant differences were observed by different letters above the graphs (ANOVA, *p* < 0.05). WT, wild type; overexpressed lines (OE-1, OE-9, and OE-10) after 8-day stress treatment. Three replications were maintained in the experiment. ANOVA, analysis of variance; SE, standard error.

In response to drought stress, we compared the physiological responses of the three transgenic lines with the wildtype. Under normal conditions, there were no significant differences between the transgenic lines and the wild type in any of the measurements, but when the plants were stressed, the transgenic lines showed greater stress tolerance than the wild type. RLWC was much higher in transgenic lines than in control lines (Figure 6I). Wild-type plants displayed more stress-induced ion leakage when they were stressed by drought. Under regulated conditions, the ELWL did not differ statistically between wild-type and transgenic plants. However, under stress conditions, the wild-type leaves lost more water than the transgenic plant leaves Figure 6II. The fact that the transformed gene improved drought stress tolerance in the transgenic Arabidopsis lines was demonstrated by the increased degree of tolerance between the transgenic lines.

### Measurements of Oxidants and Antioxidants in Transgenic and Wild-Type Arabidopsis Plants

**Figure 7:**
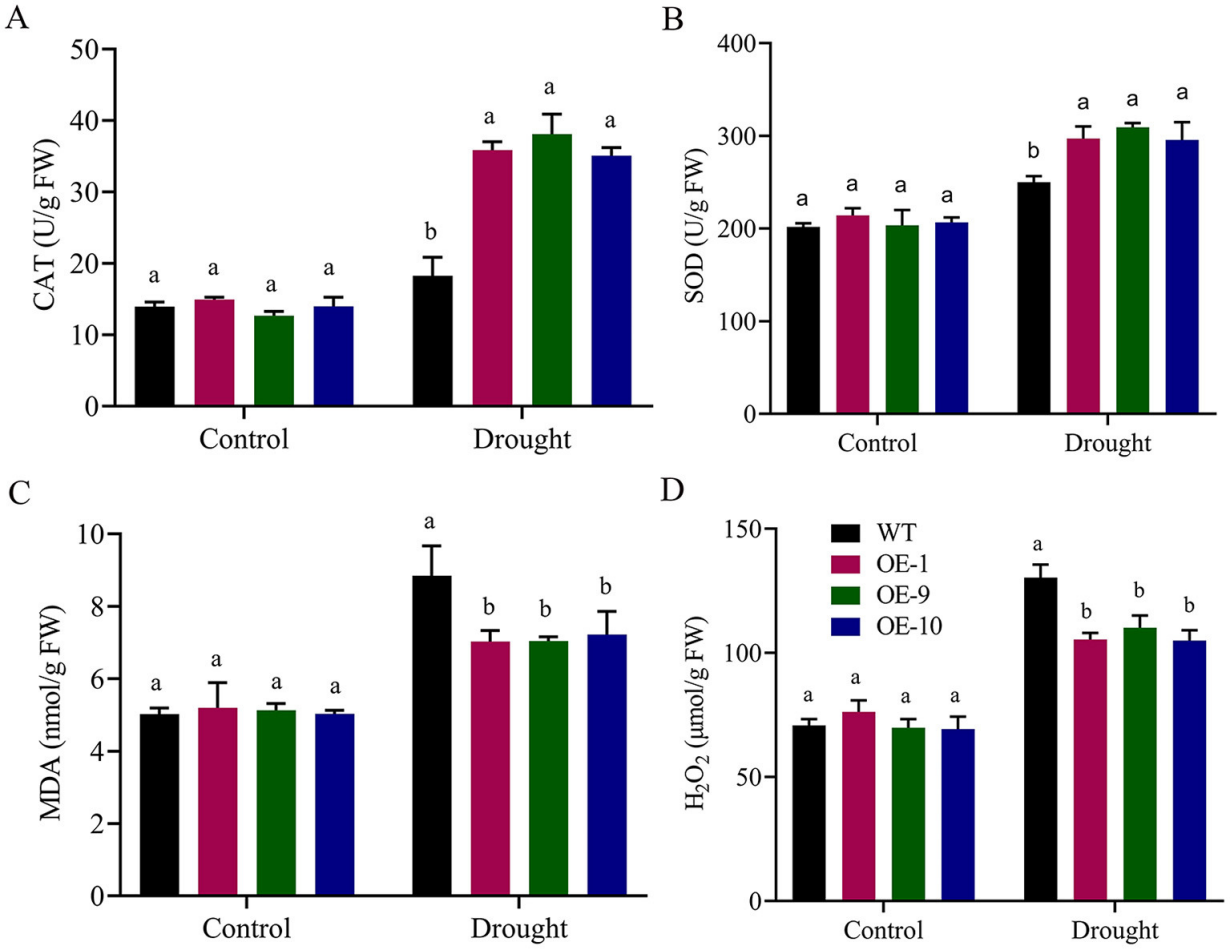
Evaluation of Antioxidants and oxidants in ALDH overexpressed and WT during drought stress **(A)** Catalase (CAT), **(B)** Superoxidase (SOD), **(C)** Malondialdehyde (MDA), **(D)** Hydrogen peroxide (H_2_O_2_) in post-treatment. Bars indicate SE. Three replications were maintained in the experiment. WT, wild type; OE-1, OE-9, OE-10, overexpressed lines. CAT, catalase; SOD, superoxidase; MDA, malondialdehyde; ALDH, Aldehyde dehydrogenase.

Activities of oxidants and antioxidants were measured in the transgenic and wild-type plants after applying drought treatment. Under control circumstances, there were no significant variations in SOD and CAT activity between transgenic lines and the wild type. However, when seedlings were exposed to drought stress after 8 days, the transgenic lines OE-1, OE-9, and OE-11 showed significant changes from the wild type, with increased SOD and CAT activity, lower MDA, and lower H_2_O_2_ levels (Figures 7A–D). Our results suggest that *Gh_A06G1257* is playing a critical role in drought stress tolerance.

### Expression Analysis of Stress-Responsive Genes

**Figure 8:**
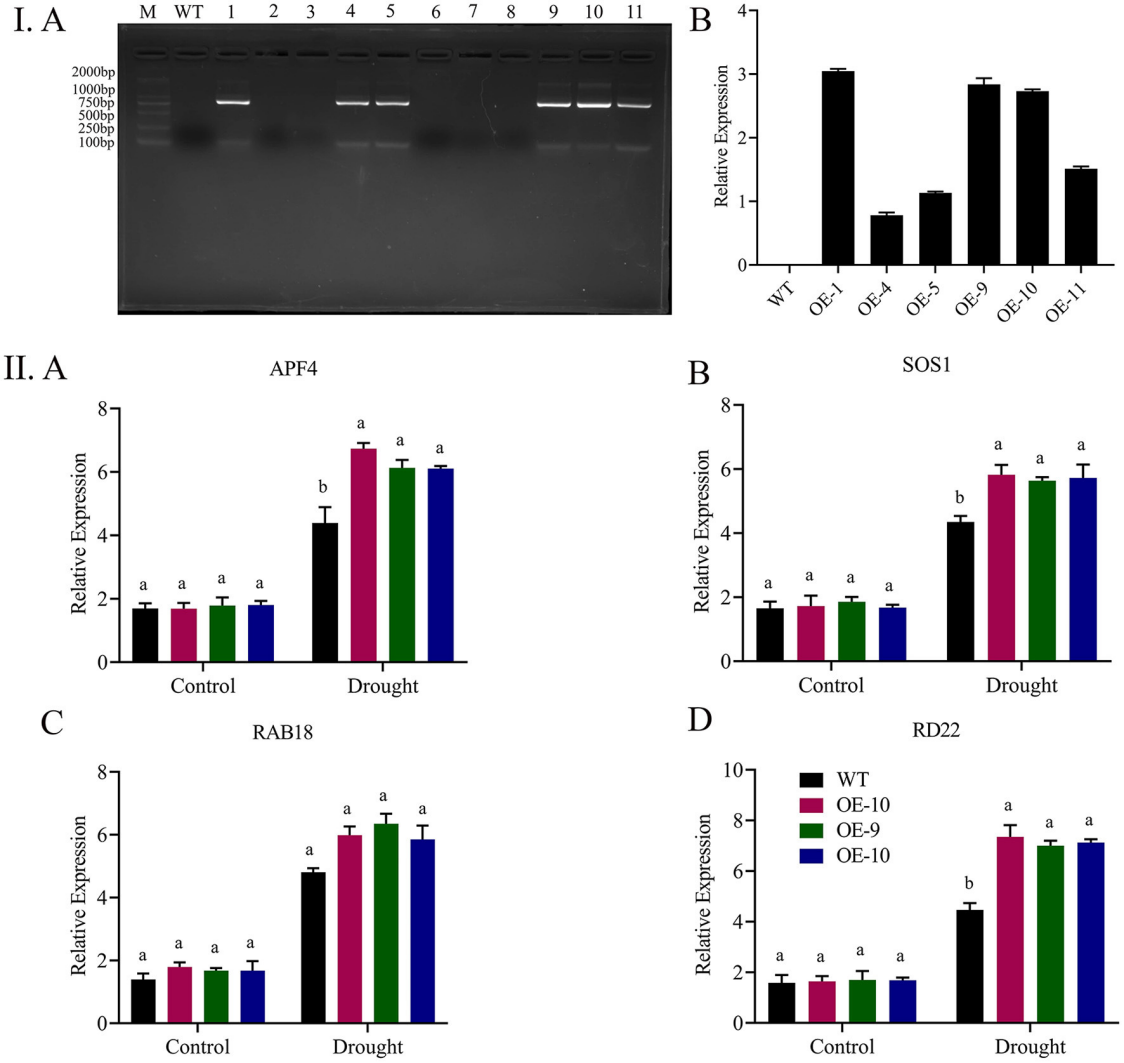
Expression analysis of Gh_A06G1257 by RT-qPCR. **(I)** A The 771 bp CDS sequence transformation to T2 generation was checked using polymerase chain reaction (PCR), 1–11 overexpressed lines, WT, wild type. B RT-qPCR was used to examine the transcript levels of the Gh A06G1257 (ALDH) of T2 overexpressed lines in three biological replications. **(II)** Expression analysis of abiotic stress-responsive genes. A APF4, B SOS1, C RAb18, D RD22 in transgenic lines (OE-1, OE-9, and OE-10) and wild type, Atactin2 gene used as an internal reference, and each experiment was done three times. CDS, coding sequence; RTqPCR, Real time quantitative polymerase chain reaction.

We choose four abiotic responsive genes ABF4, SOS1, RAB18, and RD22 to measure the expressions by qRT-PCR in over-expressed and wild-type seedlings for a better understanding of the role *Gh_A06G1257* is playing when plants are exposed to abiotic stress. Results from the RT-qPCR results revealed that upon exposure of transgenic lines to drought, the expressions of ABF4, SOS1, RAB18, and RD22 were recorded higher in the transgenic lines (Figure 8II). Thus, suggesting that *Gh_A06G1257* has a key role in coping with drought stress.

### Effects on Physio-Morphological Traits Under Drought Stress Conditions

**Figure 9:**
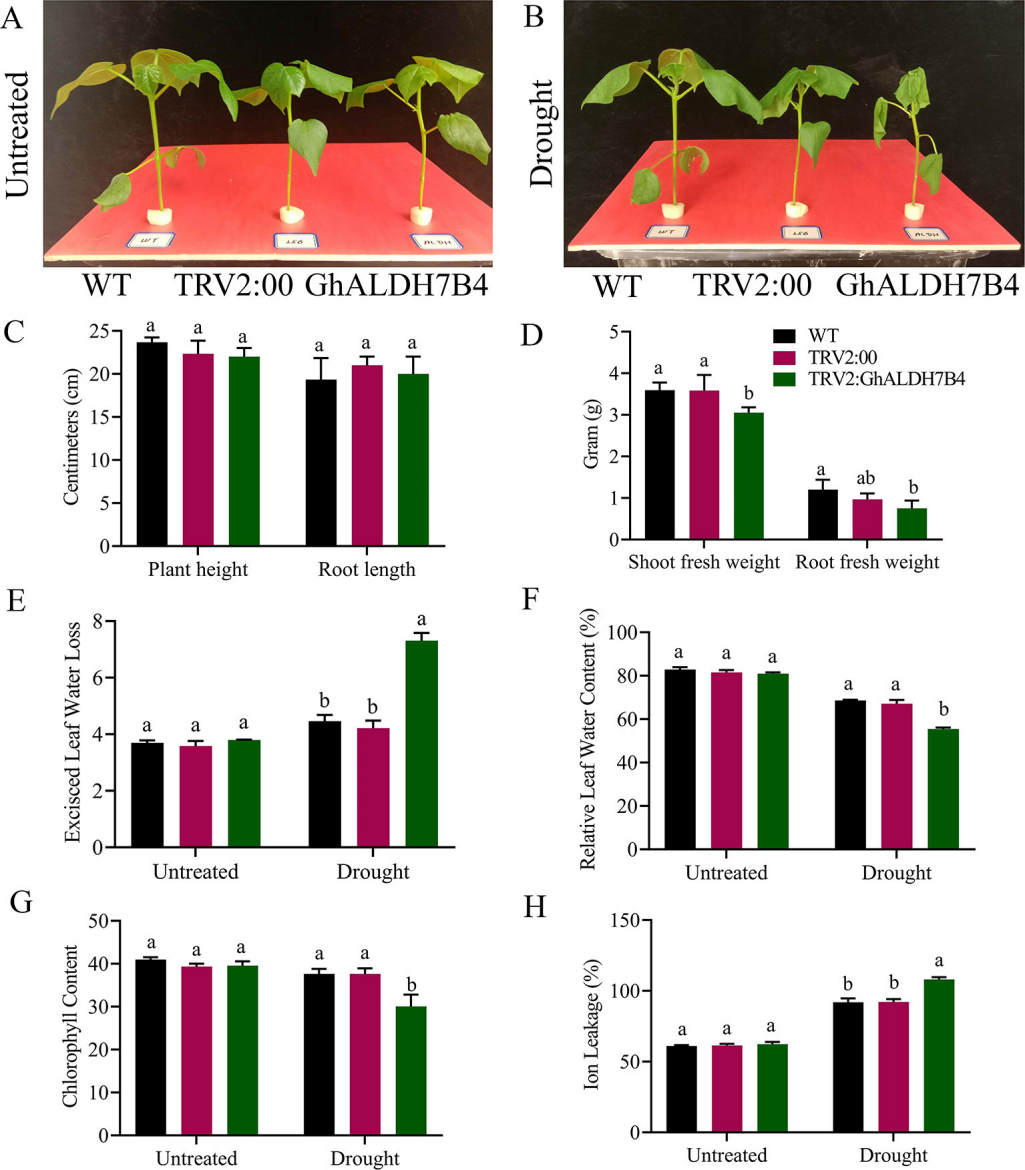
Morphological and physiological trait evaluation. **(A)** Illustrative pictures of wildtype, positive control, and silenced seedlings before stress exposure. **(B)** Illustrative pictures of wildtype, positive control, and silenced seedlings after stress exposure. **(C)** Plant height and root length. **(D)** Shoot fresh weight and root fresh weight. **(E)** Excised leaf water loss. **(F)** Relative water content, **(G)** chlorophyll content in leaves, **(H)** cell membrane stability. The error bars reflect the standard error (SE). Using the Least Significant Difference (LSD), means on the same graph, with the same letter are not significantly different at the 5% probability level, WT, wild type, TRV2:00 Empty vector, TRV2: GhALDH7B4, the silenced seedlings. Untreated: control, supplementing with 17% PEG-6000 solution. LSD, the least significant difference.

Significant variations in the physio-morphological traits of cotton were observed under drought conditions. The VIGS plants, positive controlled plants, and the wild types significantly (*p* < 0.05) differs in their response to drought stress in both morphological and physiological traits (Figure 9). Plant height and root length showed no significant differences, while VIGS plants had a considerably lower shoot and root fresh weights than wild-type pes and positive controls. Moreover, VIGS plants have a higher excised leaf water loss, and ion leakage but significantly lower relative leaf water contents than positive control and wild-type plants under drought stress.

### Measurement of Oxidants and Antioxidants in VIGS and Wild-Type Plants

**Figure 10:**
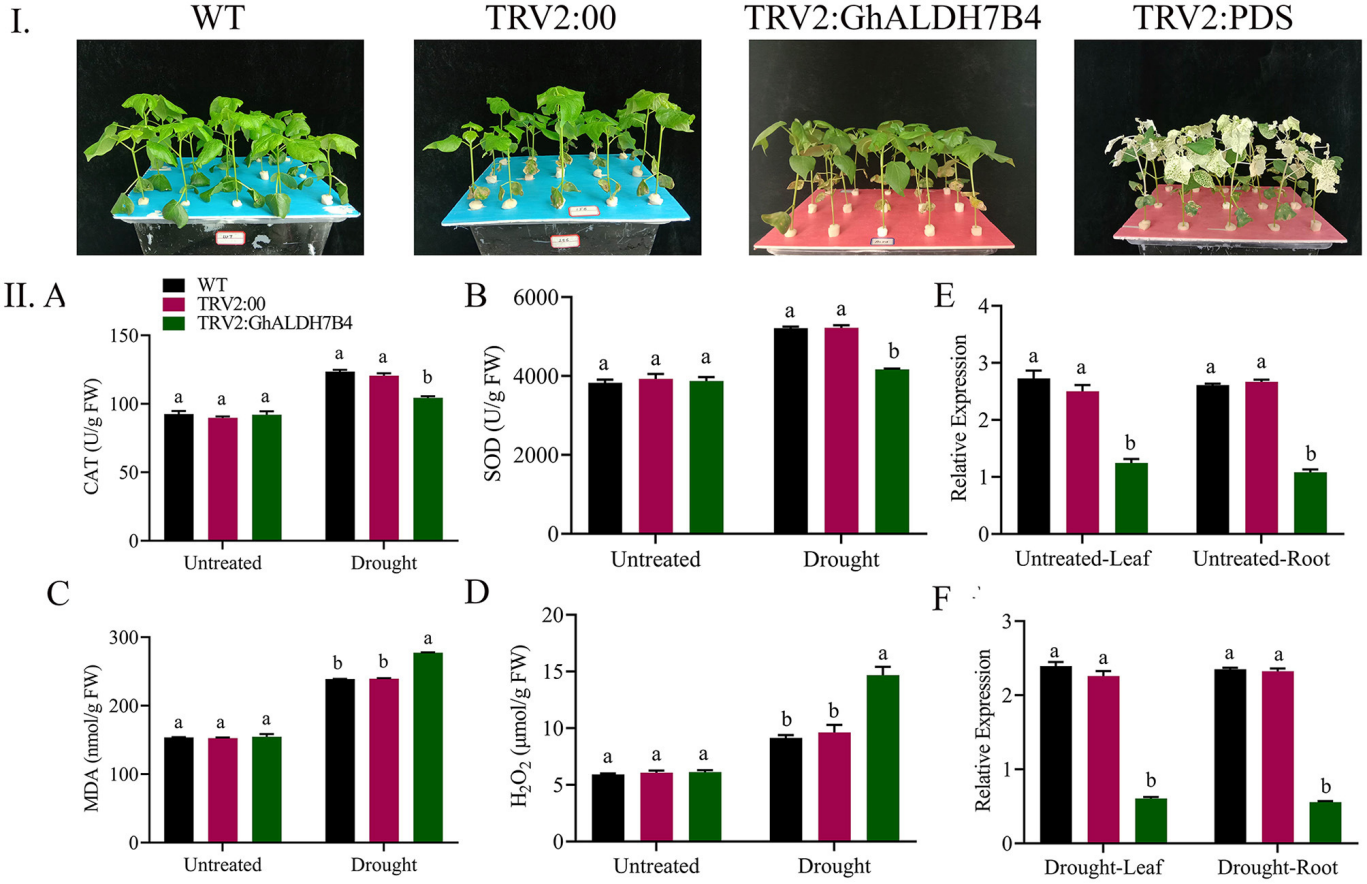
Enzyme assay and analysis of the variation in the expression levels of cotton using RT-qPCR. **(I)** Illustrative pictures of Wildtype, TRV2:00, TRV2: GhALDH7B4 and TRV2: PDS. **(II)** A Catalase, B super oxidase, C malondialdehyde, D hydrogen peroxide, E stress response gene profile in wildtype, positive control, and silenced seedlings under drought stress in the leaves. F Stress response gene profile in wildtype, positive control, and silenced seedlings under drought stress in root tissue. The error bars reflect the SE. Using the Least Significant Difference (LSD), means on the same graph, with the same letter are not significantly different at the 5% probability level. Untreated: Control, Drought: supplementing with 17% PEG-6000 solution. SE, standard error; LSD, least significant difference.

We observed significant differences (*p* < 0.001) in the oxidants and antioxidants activities among VIGS and wild-type plants. Overall, lower antioxidant activities and higher oxidant activities were measured in the case of VIGS plants (Figure 10II). CAT and SOD contents before treatment were almost the same, while after being treated with PEG-6000, the wild plants showed an increase over the VIGS plants. An increase in the contents of the oxidant enzymes activities showed that the VIGS plants experience higher oxidative stress than wild-type and positive controls. Activities of MDA and H_2_O_2_ were similar in normal conditions but recorded higher under drought stress mainly in the VIGS plants. Thus, our results suggest that the knockdown of the *Gh_A06G1257* gene has a significant influence on coping with drought stress.

### Relative Expression of the Knocked Down Gene and Wild-Type Plants

Samples from the leaves and roots of wild-type, positive control, and VIGS plants were collected. To clarify the role of the ALDH gene (*Gh_A06G1257)* under drought, we collected samples from different tissues to check the expression. The expression of *Gh_A06G1257* in VIGS plants was lower as compared to the wild-type and the positive control plants. Thus, indicating that *Gh_A06G1257* is playing a critical role in coping with drought (Figure 10IIE,F).

### Multi Pathway Enrichment Analysis

**Figure 11:**
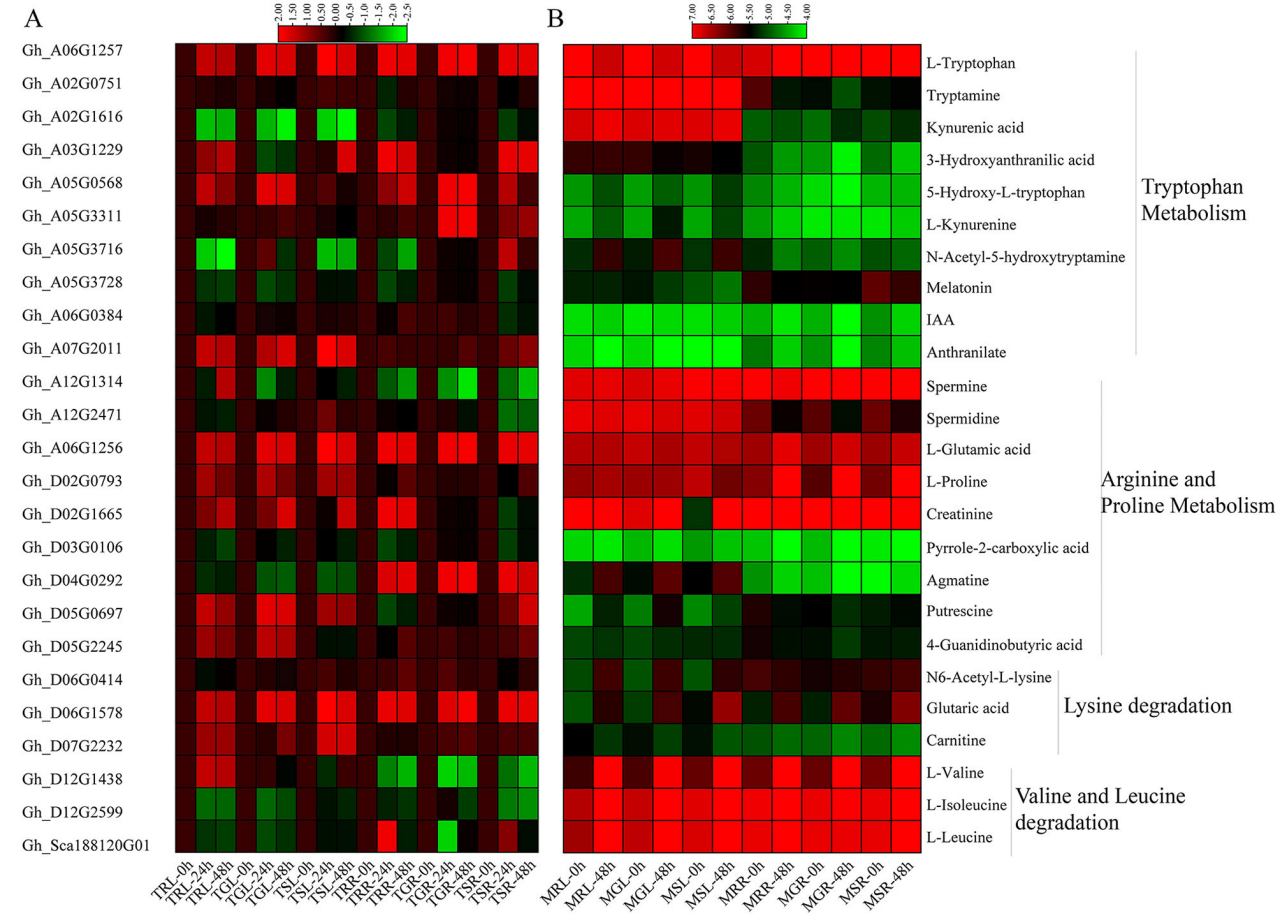
Heatmap of gene expression and expression patterns of metabolites enriched KEGG pathways. **(A)** Expression profiles of key genes involved in metabolic pathways. **(B)** The relative expression levels of metabolites based on log10 values using metabolome data in different pathways. KEGG, Kyoto encyclopedia of genes and genomes.

In the KEGG pathway annotation, the metabolites were enriched in metabolism, genetic information processing, and environmental processing information groups. A total of 25 genes playing key roles in different pathways have been identified from metabolome KEGG analysis (Supplementary Table 1B). Within this, the ALDH genes were enriched in amino acids metabolism, carbohydrates and lipids metabolism, and the replication and repair activities. The metabolism enrichment analysis found that the major enriched pathways during drought stress were arginine and proline metabolisms, tryptophan metabolism, valine and leucine degradation, and lysine degradation (Figure 11). In general, higher numbers of metabolites were upregulated in the leaves than roots under drought. In arginine and proline metabolism pathway out of nine metabolites agmatine and spermidine, in tryptophan metabolism out of ten metabolites, tryptamine, N-Acetyl-5-hydroxytryptamine, and kynurenic acid, in valine and leucine degradation mainly L-valine showed upregulation in leaf tissue. On the contrary, L-ascorbic acid in glycine, serine, and threonine metabolism, glutaric acid and N6-Acetyl-L-Lysine in lysine degradation, L-valine in valine and leucine degradation, and L-proline in arginine and proline metabolism showed upregulation in root tissues during drought stress. The rest of the metabolites showed a similar trend in both tissues.

## Discussion

Cotton, an essential industrial crop worldwide, and its production are affected by numerous stresses including both biotic and abiotic. Drought is among the key threats contributing to the significant yield losses in cotton (Hou et al., 2018). Several processes, i.e., molecular, cellular, and physiological contributes, in causing drought to result in the variations in the expression levels of genes involved in osmolyte production and their roles in improving antioxidant systems (Joshi et al., 2016). Wild relatives are believed to be a source of prominent genetic assets linked to abiotic stress tolerance (Iseki et al., 2018). Narrow genetic diversity among crops, including wild wheat, leads the cultivated species to lose tolerance to drought (Budak et al., 2013). Similarly, wild species of rice offer an extensive range of adaptive traits and can serve as potential contributors of biotic and abiotic stress tolerance (Neelam et al., 2018). In the current study, MG85 was used, which has a higher tolerance to drought and salt stresses than CRI12 (Xu et al., 2020) while LT40 is highly vulnerable to both the aforementioned stresses (Yang et al., 2019).

In total, 70,478 coding genes were present in the allotetraploid cotton *G. hirsutum* genome (TM-1) (Zhang et al., 2015). The tissues of MG85, LT40, and CRI12 were subjected to transcriptome sequencing and obtained 54 transcriptome libraries. After filtering the new data, a total of 64,617 genes were found. In a similar study, a total of 64,737 genes from 48 cDNA libraries were identified from the same test accessions under salt stress conditions (Xu et al., 2020). In distinct comparison groups, upregulated genes were considerably higher than downregulated genes in both leaves and roots. More DEGs in roots than in leaves showed that the roots are the most affected tissues under salt stress and, thus, have a multifaceted gene regulation to decrease the effect of salt stress (Xu et al., 2020). WGCNA proved to be a highly useful analysis to identify hub genes linked to drought stress tolerance in cotton.

In metabolite analysis, the principal component analysis separates the leaves and root samples, which meant that after drought treatment, the metabolites in leaves and roots respond to drought stress differently at different treatment time points. Differentially expressed metabolites analysis showed that the highest upregulated metabolites were recorded in leaves, whereas the highest downregulation of metabolites occurred in the roots. Kang et al. (2019) reported that leaves of tolerant wheat genotype changed 45 and 20 metabolites more than the roots, whereas the leaves and roots of sensitive wheat genotype changed 38 and 28 metabolites, respectively, indicating that plants dedicated more resources to the leaves of tolerant genotype. Arabidopsis plants upon grown on MS medium having supplemented mannitol, seedlings with over-expressed ALDH possess drought tolerance where wild type shows sensitivity. Our results indicate that *Gh_A06G1257* might play a key role in drought improvement during seed germination and root elongation. Moreover, overexpressed lines showed stable relative water contents, chlorophyll contents and, low water loss and ion damage as compared to the wild-type plants. We also employed pTRV-VIGS in cotton seedlings to study the part Gh*_A06G1257* is playing in drought stress. Plant height and root length showed no significant differences, while VIGS plants had a considerably lower shoot and root fresh weights than wild-type pes and positive controls. Moreover, VIGS plants have a higher excised leaf water loss, and ion leakage but significantly lower relative leaf water contents than positive control and wild-type plants under drought stress. Duan et al. (2015) studied the co-expression of the introduced bar and *CsALDH* genes in transgenic plants of Alfalfa and found that the transgenic plants retain higher relative water content levels, higher shoot biomasses, little changes in the photosystem, and reduced membrane damage.

Overexpressed plants have higher antioxidant enzymes activities that regulate the contents of ROS within the cell (Hasanuzzaman et al., 2019). Under mannitol treatment, *Gh_A06G1257* plants have higher antioxidants activities including CAT and Peroxidase (POD) whereas, lower activities of oxidant enzymes (MDA and H_2_O_2_) than wild-type (WT) plants. Under stress treatment, *GhMPK3* overexpressed plants were shown to have higher levels of antioxidants and lower contents of oxidant enzymes than WT plants (Sadau et al., 2021).

The activities of oxidant and antioxidant enzymes before the treatment were the same while after being treated with PEG-6000 solution, the wild and positive control plants showed variations over the VIGS plants. Under drought stress circumstances, the VIGS plants experienced more oxidative stress than the WT and positively controlled plants, as evidenced by the elevated levels of oxidant enzymes (Yang et al., 2019). These results were following Kirungu et al. (2020), who stated that knockdown of Dehydrin gene member meaningfully effects in reduction of plant height, root length, shoot fresh weight, and root fresh weight compared to the positive control and the wild-type plants. Similarly, contents of MDA and H_2_O_2_ were similar in normal conditions but reached high under drought stress, mainly in the VIGS plants. Plants pay well-organized detoxifying systems in response to excess-initiation of ROS under abiotic stress, containing improved activities of antioxidant enzymes such as superoxide dismutase, catalase, peroxidase, ascorbate peroxidase, and a controlled amount of non-enzymatic antioxidants such as glutathione and ascorbic acid (Shi et al., 2015; Ding et al., 2018). Therefore, crops must preserve the amount of ROS at a proper level under abiotic stresses.

According to our results, *Gh_A06G1257* was found to be a candidate gene responsible for tolerance to drought stress. Results from VIGS in cotton and overexpression in Arabidopsis also confirm the role this gene is playing to cope with drought. An over-expression research in *A. thaliana* of the *VvALDH2B4* gene boosted protection against high salt and pathogenic bacteria and bring about lower MDA levels (Wen et al., 2012). When *ScALDH21* was introduced into tobacco plants, it resulted in greater germination ratios, root lengths, proline concentrations, antioxidant enzyme actions, and decreased MDA levels (Yang et al., 2015). *CaALDH1* gene silencing in pepper disturbed phenolic compound buildup, H_2_O_2_ production, protection response gene expression, and cell death, while in transgenic *A. thaliana*, overexpressing *CaALDH1* revealed improved defense response to infection (Kim and Hwang, 2015).

*BrALDH7B2* has the potential to cope in both abiotic stress and hormonal treatments in *Brassica rapa*. Over-expressing *BrALDH7B2* in *E. coli* and yeast cells resulted in a substantial tolerance to abiotic stress. Hence, *BrALDH* genes need to be explored more as they play a key role to cope with abiotic stress in *B. rapa* (Gautam et al., 2019). Drought stress in soybean leaves strongly supported the functions of *GmALDH* genes, including *GmALDH3H2, GmALDH12A2*, and *GmALDH18B3* (Wang et al., 2017). The expression of “*ALDH3I1”* and “*ALDH7B4”* genes was adequate to boost tolerance against drought, salinity, and oxidative stress in crops (Kotchoni et al., 2010). Plant *ALDH7B* expression has been found to be responsive to a wide range of stressors in studies, and expression is assumed to be a part of general stress-response pathways. UV exposure, dehydration, high salinity, low temperature, heat shock, and applied behavior analysis (ABA) therapy are just some of the stressors that cause *ALDH7B* overexpression (Brocker et al., 2013).

In the KEGG pathway annotation, ALDH genes play key roles in different pathways that have been identified as the home of metabolites during drought stress. L-ascorbic acid, agmatine, spermidine, myoinositol, ectoine, tryptamine, N-Acetyl-5-hydroxytryptamine, and kynurenic acid in leaf tissue, glutaric acid, L-ascorbic acid, N6-Acetyl-L-Lysine, L-tryptophan, and L-proline in root tissues showed upregulation. The ALDH gene family, which is involved in many pathway regulations and enzymes, appears to play a primary role in abiotic stress-response pathways. These metabolites could be an important target for increasing plant resistance to stressful conditions like elevated soil salinity or dehydration, which is especially important when developing stress-tolerant crops (Brocker et al., 2013). Metabolites like proline, L-arginine, L-histidine, L-isoleucine, and tryptophan exhibited increased after drought stress, which was likely the sign of acclimation in response to drought stress in chickpeas (Khan et al., 2019). Similarly, in maize research, an increased amount of proline was observed under drought stress and tryptophan addition increased Indole-3-acetic acid (IAA) and Gibberellic acid (GA) production, and further increased Abscisic acid (ABA) production in water shortage areas, demonstrating that tryptophan addition may help enhance drought tolerance (Yasmin et al., 2017).

Plants produce large amounts of ROS under drought stress. Most of these substances are antioxidants that can increase the drought resistance of plants under drought stress. Excessive amounts of these ROS can cause damage to plant cells and can lead to plant death (Suzuki et al., 2012; Baxter et al., 2014; Nakabayashi et al., 2014; An et al., 2015). Plants, sensing the osmotic stress through their receptor, will induce the regulation and expression of genes in the body and generate a large amount of metabolism through various amino acids, lipids, carbohydrates, and secondary metabolism. Antioxidant substances such as glutathione, lysine, proline, ascorbic acid, terpenoids, and flavonoids respond to drought stress (Nakabayashi et al., 2014; Wang et al., 2016). Similarly, a prominent group of metabolites affected by drought stress is amino acids, organic acids, sugars, sugar alcohol, and fatty alcohol. Tolerant genotypes distributed more resources to leaves than roots, whereas sensitive genotypes allocated resources to roots and leaves in a similar way (Kang et al., 2019). Tryptophan is an osmolyte that regulates ion transport, modulates stomatal opening, and maintains water balance (Rahman et al., 2017). Proline has been positively correlated with stress tolerance, serves as a compatible solute, and helps plants to avoid oxidative stress by keeping reduced levels of ROS (Hayat et al., 2012). Raised tryptophan and proline was crucial in drought stress condition (Kang et al., 2019). Similarly, an increase in lysine concentration encourages the transcriptional upregulation of genes enhancing lysine-to-a-aminoadipate metabolic instability and using glutamate to yield proline to respond to abiotic and biotic stress (Arruda and Barreto, 2020). In the RNA-Seq and RT-qPCR analysis, most genes were upregulated thus, demonstrating that these genes could be playing a significant role in enhancing drought stress tolerance in cotton. Similarly, *Gh_A06G1257* was found to be upregulated in all species and time points according to RNA-Sequencing as well as RT-qPCR analysis, which revealed that this is a true potential candidate for drought stress tolerance in cotton. Cai et al. (2019) stated that RT-qPCR results were significantly correlated to the RNA-Seq data both at 6 and 12 h points under cold stress (*r*^2^ = 0.88 and 0.95) in *G. thurberi*.

## Conclusion

In the current research work, an integrated transcriptome and metabolome approach to investigate gene/s to cope with drought in cotton was utilized. Thirteen hub genes were initially identified by WGCNA. Further expression analysis in both leaves and root tissues revealed that *Gh_A06G1257* (*GhALDH7B4*) belonging to the ALDH family might be the candidate gene. We opted for VIGS and overexpression approaches for functionally validating the candidate gene and current results suggest that *Gh_A06G1257* is involved in drought stress tolerance in cotton. L-valine, Glutaric acid, L-proline, L-Glutamic acid, and L-Tryptophan were found to be the upregulated metabolites in the pathway annotation. This gene is newly identified. No previous reports regarding its role or functions in cotton are available. The results of this study may add to a profound understanding of the highly multifaceted genes and regulatory mechanisms that function in plants during drought stress.

## Data Availability Statement

The datasets presented in this study can be found in online repositories. The names of the repository/repositories and accession number(s) can be found below: NCBI (accession: PRJNA663204).

## Author Contributions

TM: conceptualization, methodology, and writing original draft. YX: conceptualization and methodology. MU: writing—review and editing. MS: formal analysis and investigation. YH, YW, SY, and XZ: investigation and resources. KW, XC, and ZZ: software and validation. FL: supervision. All authors contributed to the article and approved the submitted version.

## Funding

This research was funded by the National Natural Science Foundation of China (31621005 and 32072023), Agricultural Science and Technology Innovation Program of the Chinese Academy of Agricultural Sciences.

## Conflict of Interest

The authors declare that the research was conducted in the absence of any commercial or financial relationships that could be construed as a potential conflict of interest.

## Publisher's Note

All claims expressed in this article are solely those of the authors and do not necessarily represent those of their affiliated organizations, or those of the publisher, the editors and the reviewers. Any product that may be evaluated in this article, or claim that may be made by its manufacturer, is not guaranteed or endorsed by the publisher.
