# Supplementary material for: Multi-Omics-Based Identification and Functional Characterization of Gh_A06G1257 Proves Its Potential Role in Drought Stress Tolerance in Gossypium hirsutum
Source: Front Plant Sci. 2021 Oct 21;12:746771. doi: 10.3389/fpls.2021.746771 (PMC8567990; doi:10.3389/fpls.2021.746771)
Supplement: Supplementary file 1 [file Data_Sheet_1.zip › Supplementary Captions.doc]

**Supplementary Captions**

**Supplementary Figure 1** l Gene Ontology Analysis of Cotton semi wild lines, (**A**) Biological function of leaves in MG85 (**B**) Molecular function of leaves in MG85 (**C**) Biological function of roots in MG85 (**D**) Biological function of leaves in CRI12 (**E**) Molecular function of leaves in CRI12 (**F**) Biological function of roots in CRI12 (**G**) Cellular function of roots in CRI12 (**H**) Biological function of leaves in LT40 (**I**) Cellular function of roots in LT40 (**J**) Molecular function of leaves in LT40.

**Supplementary Table 1 l** Primer design details of RTq-PCR for ALDH genes

**Supplementary Table 2 l** Pathway annotation for differentially expressed metabolites in leaf and root tissues of the tested materials

**Supplementary Table 3 l** List of differentially expressed genes in leaf and root tissue of the three accessions in different time points

**Supplementary Table 4 l** List of metabolite Pathways with their gene and metabolite expression

**Supplementary Table 5 l** List of genes used to analyze the coexpression network analysis from brown, pink and yellow modules
